# Anxiety as signal: a practitioner framework for reframing music performance anxiety

**DOI:** 10.3389/fpsyg.2026.1884849

**Published:** 2026-07-22

**Authors:** Donald M. Goodman

**Affiliations:** Restorative Performance and Wellness, Westlake Village, CA, United States

**Keywords:** arousal reappraisal, autonomic nervous system, cognitive appraisal, Goodman method, music performance anxiety, performance coaching, polyvagal theory

## Abstract

Music performance anxiety (MPA) is among the most prevalent challenges faced by musicians across training and professional contexts. Prevailing clinical and pedagogical approaches frame MPA primarily as a dysfunction that needs to be reduced, producing interventions that often target symptom suppression rather than the underlying cognitive appraisal processes that generate the meaning of anxiety. This perspective article proposes an alternative framework: the physiological activation accompanying high-stakes performance constitutes adaptive signaling, not pathological malfunction, and durable performance gains accrue not from reducing arousal but from reappraising its meaning. Drawing on autonomic nervous system science, cognitive appraisal theory, and two decades of practice spanning concert piano performance and clinical psychology, the author describes the Goodman Method®—a practitioner-applicable framework comprising four evidence-grounded techniques: arousal reappraisal, curiosity-based attentional redirection, coherent breathing for parasympathetic activation, and performance meaning reconstruction. The framework is positioned explicitly in relation to existing centering and Acceptance and Commitment Therapy approaches, with which it shares substantial conceptual similarities. Implications for music educators, performance coaches, and clinicians treating MPA are discussed.

## 1 Introduction

Music performance anxiety (MPA) is defined as persistent, distressing apprehension related to musical performance that impairs functioning or undermines subjective experience (Kenny, 2011). Prevalence estimates across professional and conservatory-level musicians typically range from 25% to over 70%, depending on the severity threshold and population studied (Papageorgi et al., 2011; Mor et al., 1995). Despite decades of research, MPA remains substantially undertreated in applied music settings, and the interventions most readily available to practitioners—breathing exercises, positive self-talk, and beta-blocker pharmacology—share a common conceptual foundation: anxiety as a problem, and its reduction as the goal.

This framing, while clinically familiar, may be incomplete. The recognition that anxiety, including MPA specifically, may function adaptively rather than purely as pathology is well established in the existing literature (Barlow, 2002; Wolfe, 1989; Osborne and Kirsner, 2022). The de-pathologizing of MPA as a signal has been articulated directly in the music psychology literature (Herman and Clark, 2023), and the present article builds on this foundation rather than introducing it. This article argues that the physiological substrate of MPA, which includes sympathetic nervous system activation, heightened arousal, and attentional narrowing, is not inherently maladaptive. These responses evolved as functional preparatory systems; they become problematic primarily when cognitively appraised as evidence of threat rather than readiness. From this perspective, the primary intervention target should not be arousal itself, but the interpretive schema through which performers assign meaning to arousal.

The following sections describe the theoretical foundations of this framework and operationalize them through four practitioner-applicable techniques grounded in peer-reviewed science and refined through two decades of clinical and performance coaching practice. The author occupies a dual position—concert-trained pianist and clinical psychologist—that informs the framework’s integration of performer-insider knowledge with psychological science.

## 2 Theoretical foundations

### 2.1 Autonomic activation is not pathology

The physiological signature of MPA—elevated heart rate, increased muscle tension, heightened alertness, peripheral vasoconstriction—is indistinguishable from the autonomic response to any high-salience event (McEwen, 1998). Porges’ polyvagal theory (2011) offers a nuanced account: the autonomic nervous system operates across a hierarchy of engagement states, with sympathetic mobilization representing not dysfunction but a functional preparation for demands requiring heightened output. In performance contexts, this mobilization is appropriate.

The problem arises not from the activation itself, but from its appraisal. When performers interpret racing pulses and trembling hands as evidence of impending failure, they trigger a secondary fear response—fear of the fear—that compounds arousal and disrupts attentional and motor systems (Barlow, 2002). Suppression attempts typically exacerbate this dynamic: Effortful attempts to “calm down” consume the cognitive resources needed for performance and signal to the self that the internal state is indeed dangerous (Wegner, 1994).

### 2.2 Cognitive appraisal and the arousal–performance relationship

Lazarus and Folkman (1984) cognitive appraisal theory proposes that emotional responses are not direct products of events but of the meanings attributed to them. Applied to MPA, this framework predicts that the same arousal state can function as facilitating or debilitating depending on the performer’s appraisal. This facilitating/debilitating distinction has been a specific focus of empirical studies in music performance (Simoens et al., 2015; Wolfe, 1989). This prediction has received broader empirical support: Jamieson et al. (2010) demonstrated that reappraising physiological arousal as “excitement” rather than “anxiety” improved performance on cognitive tasks under stress, accompanied by corresponding changes in cardiovascular profiles. Cheng et al. (2009), extending this finding to music performance contexts specifically, showed that reappraisal instructions reduced debilitating anxiety and improved performance quality relative to control conditions. Osborne and McPherson (2019) extended this line of work further in conservatorium musicians, testing Lazarus’s (1999) cognitive-motivational-relational model of primary and secondary appraisal in relation to facilitating and debilitating anxiety, confidence, and objective performance outcomes. Performers who appraised an upcoming recital as a challenge rather than a threat achieved exam marks roughly two grade categories higher on average, providing a valuable empirical bridge between appraisal and measured performance quality.

Importantly, these effects were not produced by reducing arousal—physiological activation remained elevated. The mechanism was cognitive: performers’ interpretive relationship to their own bodily states shifted, and performance followed. This distinction is theoretically significant. It suggests that the target of intervention should be the appraisal system rather than the autonomic system itself.

### 2.3 Curiosity as antidote to catastrophizing

A distinctive feature of anxiety is its tendency toward catastrophic elaboration: the initial signal of the nervous system is amplified by a cascade of worst-case interpretations. Kashdan and Silvia (2009) describe curiosity as a functionally antagonistic motivational state—one that redirects attention from threat-monitoring toward open-ended exploration, interrupting the amplification cycle. In performance coaching contexts, cultivating a curious relationship with internal experience—asking “What is my body telling me?” rather than “What is wrong with me?”—is a tractable, low-cost intervention that can be implemented during sessions and practiced outside of formal treatment.

This perspective is also consistent with mindfulness-based approaches to music performance anxiety, which encourage non-judgmental observation of internal experience rather than attempts at suppression or control (Kabat-Zinn, 1990; Khalsa et al., 2009; Diaz, 2018). Curiosity, as conceptualized here, is not proposed as a novel intervention but as an accessible operationalization of mindfulness principles within performance settings. It is also closely aligned with Acceptance and Commitment Training approaches developed specifically for musicians, which similarly emphasize non-judgmental, present-moment awareness of internal experience as a foundation for performance (Juncos and de Paiva e Pona et al., 2017; Juncos and de Paiva e Pona, 2022). By shifting attention from evaluative judgment to observation and inquiry, performers may develop greater psychological flexibility in relation to physiological arousal and performance-related thoughts.

### 2.4 A practitioner framework: four evidence-grounded techniques

The proposed framework is a practitioner model for reframing MPA as an adaptive signal (Goodman, 2025). It comprises four techniques, each targeting a distinct mechanism in the anxiety-performance cascade. All four can be taught in applied music instruction, performance coaching, or brief clinical consultation without requiring formal psychotherapy training.

#### 2.4.1 Arousal reappraisal: renaming the experience

The foundational intervention is linguistic and cognitive: teaching performers to describe their pre-performance arousal as activation rather than anxiety. This reframe is not positive thinking—it does not deny the physiological state. It accurately reattributes the state’s meaning. “Your nervous system is preparing you” is a true statement, and its accuracy matters: performers who find the reframe credible internalize it more durably than those who receive empty reassurance.

In practice, this involves explicitly naming the physiological symptoms before performance and reattributing them: “The racing heart you feel is increased cardiac output. The heightened alertness is your attentional system focusing. This is your body doing its job.” This language can be used by teachers, coaches, and clinicians without clinical training and can be introduced to young performers with developmentally appropriate vocabulary. This specific reframe is not unique to the present framework: Greene (2002) similarly instructs performers to relabel heightened pre-performance states as “activation” rather than “nervousness” (p. 31), and comparable language appears in music-specific pedagogical contexts (Osborne and Kirsner, 2022, p. 222; Osborne, 2023, pp. 87–90). The contribution of the present framework lies not in this individual element but in its integration with the curiosity-based, breath-based, and meaning-focused techniques that follow, within a single sequential protocol suited to studio and coaching contexts.

#### 2.4.2 Curiosity-based attentional redirection

When a performer catastrophizes, attention narrows to the feared outcome and away from the performance itself. The antidote is curiosity—specifically, deliberate attention to sensory and somatic experience without an evaluative overlay. Instructors can introduce this with a simple shift in language: replacing “Are you nervous?” (which invites binary, evaluative self-report) with “What do you notice in your body right now?” (which invites observational self-awareness).

This shift accomplishes two things. First, it models a non-evaluative relationship with internal experience. Second, it moves the performer from being inside the fear—fused with it—to observing it, creating the psychological distance that interrupts amplification. This approach is consistent with mindfulness-based interventions that emphasize present-moment awareness and non-judgmental observation of thoughts, emotions, and bodily sensations. Curiosity serves as a practical attentional strategy for implementing these principles in performance settings.

#### 2.4.3 Coherent breathing for parasympathetic activation

Slow, rhythmic breathing at approximately five to six breath cycles per minute—termed coherent breathing by Lehrer and Gevirtz (2014)—reliably activates the parasympathetic nervous system via vagal afferent pathways, increases heart rate variability, and reduces sympathetic arousal. This technique requires no pharmacological intervention, specialized equipment, or extensive training, and its effects are immediate. Slow-paced breathing protocols for anxiety and stress reduction are well established in the broader clinical literature, with a recent systematic review synthesizing implementation guidelines across this evidence base (Bentley et al., 2023). Within music pedagogy specifically, comparable square and rectangular-breathing protocols for somatic arousal management have been described by Osborne and Kirsner (2022, pp. 220–221).

The practical protocol is straightforward: inhale for a count of five, exhale for a count of five, and repeat three to five times before performance. Porges (2011) notes that this breathing pattern directly engages the ventral vagal circuit associated with social engagement and calm readiness, which is precisely the physiological state optimal for performance. When taught as a consistent pre-performance ritual, this technique becomes automatic and serves as a reliable anchor under pressure.

#### 2.4.4 Performance meaning reconstruction

Much of the MPA is rooted in the implicit framing of performance as a verdict—a moment of judgment determining whether the performer is adequate. This framing maximizes the stakes and minimizes self-compassion. An alternative framing positions performance as communication: an opportunity to share preparation, artistry, and musical intention with others.

This perspective does not deny that many performance situations are inherently evaluative. Auditions, competitions, juries, and professional screenings often involve explicit judgment, limited opportunities, and meaningful career consequences. In such settings, excellence is a legitimate goal, and performance outcomes are important. The purpose of meaning reconstruction is therefore not to minimize the realities of evaluation but to broaden the performer’s relationship with it. Musicians can pursue excellence while maintaining a sense of self-worth that is not wholly dependent on a single outcome. Self-compassion and evaluative rigor need not be mutually exclusive.

Meaning reconstruction draws on Frankl’s (2006) observation that the human capacity to choose one’s interpretive relationship to an experience is the final unassailable freedom. Practically, this means helping performers articulate a purpose for each performance that transcends technical execution: “Your job tonight is to share this music” rather than “Your job tonight is to not make mistakes.”

### 2.5 Relationship to centering and acceptance and commitment approaches

The four techniques described above share substantial conceptual ground with two established approaches in performance psychology: centering, as developed by Greene (2002, 2021) and applied in music training contexts by Osborne et al. (Osborne et al., 2014; Osborne et al., 2022; Osborne and Kirsner, 2022; Osborne, 2023), and Acceptance and Commitment Therapy/Training (ACT) as adapted for musicians (Juncos and de Paiva e Pona et al., 2017; Juncos and de Paiva e Pona, 2022; Juncos et al., 2017).

Centering combines attentional control and cognitive restructuring—refocusing attention toward the desired performance outcome—with physiological regulation through breath work and tension release, treating performance arousal as valuable performance energy rather than a state to be eliminated. This overlaps directly with the arousal reappraisal and coherent breathing techniques described above; notably, centering protocols also include upregulating instructions to achieve optimal physiological activation when performers are under-aroused (Greene, 2002, pp. 98–100), a dimension not addressed in the present framework and a direction for future development.

ACT approaches for musicians combine mindfulness-based, non-judgmental attention to internal experience with value-oriented action—a combination that maps onto the curiosity-based attentional redirection and performance meaning reconstruction techniques described above (Juncos and de Paiva e Pona et al., 2017; Juncos and de Paiva e Pona, 2022).

Given these substantial overlaps, the present framework does not claim originality in individual techniques. Its intended contribution is narrower: a brief, sequential, practitioner-facing protocol—arousal reappraisal, curiosity, breath, meaning—that can be taught by music educators and performance coaches without clinical training, drawing together established elements of centering and ACT into a form suited to brief studio or coaching contexts rather than extended clinical or sport-psychology training programs. Readers and practitioners seeking fuller treatments of these approaches are encouraged to consult the centering and ACT literature cited above directly.

## 3 Discussion

The framework proposed here represents a shift in emphasis rather than a wholesale departure from existing MPA intervention literature. Cognitive-behavioral approaches to MPA (Kendrick et al., 1982; Clark and Agras, 1991) have demonstrated efficacy and remain the most extensively validated psychotherapeutic interventions. Acceptance and Commitment Therapy (ACT) approaches, which share the non-suppression orientation of the present framework, have shown promising results in recent trials (Juncos et al., 2017). The Goodman Method® is complementary to both: It targets the earliest point in the anxiety cascade (appraisal of arousal) and can be implemented before formal clinical intervention is warranted. This practitioner-level, non-clinical entry point is consistent with recent calls to equip educators and coaches without clinical specialization to support performers’ self-efficacy in directly managing the MPA signal (Gill et al., 2026)^1^.

A limitation of the present framework is the relative absence of controlled empirical validation of these four techniques as an integrated system. Each component rests on a substantial evidence base from adjacent literatures—appraisal theory, polyvagal science, curiosity research, meaning-making psychology—but the Goodman Method® as a packaged intervention has not yet been subjected to randomized controlled evaluation. The author’s doctoral research (Goodman, 2005) demonstrated that autonomic nervous system-targeted interventions produced durable MPA reductions in solo classical pianists, providing preliminary support for the ANS-focused components; broader empirical validation of the integrated framework is a priority for future research.

The practitioner implications are considerable. Music teachers, performance coaches, athletic coaches, and educators working with anxious performers need not wait for a formal clinical referral to introduce these techniques. Arousal reappraisal, curiosity cultivation, coherent breathing, and meaning reconstruction are teachable, non-clinical, and grounded in peer-reviewed scientific research. Their integration into music pedagogy and performance coaching represents a practical expansion of the field’s capacity to address MPA where it first presents—in the studio, in the wings, and at the piano.

## 4 Conclusion

Performance anxiety is not necessarily a malfunction to be eliminated, but a signal arising from the interaction of biological threat-detection systems, contextual demands, personal meaning, and individual appraisal processes. The capacity to respond to that signal with curiosity rather than fear, to interpret activation as readiness rather than danger, and to find meaning in the act of performance itself is a learnable skill rather than a fixed trait.

The Goodman Method® operationalizes this reframe across four evidence-grounded techniques accessible to practitioners without clinical specialization, developed in explicit dialogue with—rather than in place of—the established centering and ACT literature described above. Future controlled research should examine the method’s efficacy as an integrated intervention protocol. Until such evidence accrues, the framework offers a theoretically coherent, practically accessible alternative to suppression-based approaches—one that begins with a simple premise: anxiety need not be viewed as the enemy of performance.

## Footnotes

1 Gill, A., Osborne, M. S., & McPherson, G. E. (2026). Building confidence in music performance: a teacher-led self-efficacy approach. *Music Education Research*, 1–21. https://doi.org/10.1080/14613808.2026.2672352
